# Multi-model quantitative MRI of uterine cancers in precision medicine’s era—a narrative review

**DOI:** 10.1186/s13244-025-01965-z

**Published:** 2025-05-28

**Authors:** Marco Gennarini, Rossella Canese, Silvia Capuani, Valentina Miceli, Federica Tomao, Innocenza Palaia, Valentina Zecca, Alessandra Maiuro, Ilaria Balba, Carlo Catalano, Stefania Maria Rita Rizzo, Lucia Manganaro

**Affiliations:** 1 Department of Radiological, Oncological and Pathological Sciences, Umberto I Hospital, “Sapienza” University of Rome, Rome, Italy; 2 Core Facilities, Istituto Superiore di Sanità, Viale Regina Elena 299, Rome, Italy; 3 National Research Council (CNR), Institute for Complex Systems (ISC) c/o Physics Department Sapienza University of Rome, Rome, Italy; 4 Department of Maternal and Child Health and Urological Sciences, Sapienza University of Rome, Rome, Italy; 5 Department of Basic and Applied Sciences for Engineering, University of Rome Sapienza, Rome, Italy; 6 Siemens Healthcare S.r.l., dARE, Milan, Italy; 7 Istituto di Imaging della Svizzera Italiana (IIMSI), Ente Ospedaliero Cantonale (EOC), Lugano, CH Switzerland; 8 Facoltà di Scienze Biomediche, Università della Svizzera Italiana, Lugano, CH Switzerland

**Keywords:** Magnetic resonance imaging, Uterine neoplasms, Contrast media, Diffusion magnetic resonance imaging, Spectrum analysis

## Abstract

**Purpose:**

This review aims to summarize the current applications of quantitative MRI biomarkers in the staging, treatment response evaluation, and prognostication of endometrial (EC) and cervical cancer (CC). By focusing on functional imaging techniques, we explore how these biomarkers enhance personalized cancer management beyond traditional morphological assessments.

**Methods:**

A structured search of the PubMed database from January to May 2024 was conducted to identify relevant studies on quantitative MRI in uterine cancers. We included studies examining MRI biomarkers like Dynamic Contrast-Enhanced MRI (DCE-MRI), Diffusion-Weighted Imaging (DWI), and Magnetic Resonance Spectroscopy (MRS), emphasizing their roles in assessing tumor physiology, microstructure, and metabolic changes.

**Results:**

DCE-MRI provides valuable quantitative biomarkers such as Ktrans and Ve, which reflect microvascular characteristics and tumor aggressiveness, outperforming T2-weighted imaging in detecting critical factors like myometrial and cervical invasion. DWI, including advanced models like Intravoxel Incoherent Motion (IVIM), distinguishes between normal and cancerous tissue and correlates with tumor grade and treatment response. MRS identifies metabolic alterations, such as elevated choline and lipid signals, which serve as prognostic markers in uterine cancers.

**Conclusion:**

Quantitative MRI offers a noninvasive method to assess key biomarkers that inform prognosis and guide treatment decisions in uterine cancers. By providing insights into tumor biology, these imaging techniques represent a significant step forward in the precision medicine era, allowing for a more tailored therapeutic approach based on the unique pathological and molecular characteristics of each tumor.

**Critical relevance statement:**

Biomarkers obtained from MRI can provide useful quantitative information about the nature of uterine cancers and their prognosis, both at diagnosis and response assessment, allowing better therapeutic strategies to be prepared.

**Key Points:**

- Quantitative MRI improves diagnosis and management of uterine cancers through advanced imaging biomarkers.
- Quantitative MRI biomarkers enhance staging, prognosis, and treatment response assessment in uterine cancers.
- Quantitative MRI biomarkers support personalized treatment strategies and improve patient management in uterine cancers.

**Graphical Abstract:**

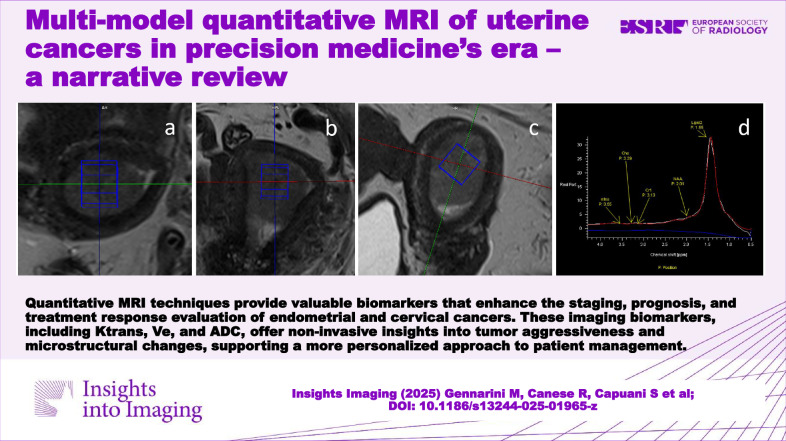

## Introduction

Biomarkers are features that can be objectively measured and evaluated as indicators of normal biological processes, pathogenic processes, or pharmacological responses to a therapeutic intervention. The final goal of finding tumoral biomarkers is to achieve a more personalized management based not only on the extent of the disease but also on its pathological and molecular characteristics.

In the last few decades, important developments in imaging techniques have led to significant improvements in tumor staging, monitoring, and early detection of disease relapses. In particular, recent imaging research has focused on the assessment of functional tissue characteristics, leading to an updated diagnostic approach of radiologists, which was previously based only on morphologic features, and now relies also on functional imaging methods.

Furthermore, imaging examinations offer an exceptional opportunity to evaluate quantitative features from data mining of examinations routinely performed in cancer patients, such as ultrasound (US), computed tomography (CT), and magnetic resonance imaging (MRI), as demonstrated by the increasing number of publications related to quantitative biomarkers, including technical evaluations [1–3], radiomics/radiogenomics [4, 5], and the wide world of artificial intelligence-based models [6, 7].

Epithelial tumors of the uterus may originate from either cervix and corpus with different etiopathogenesis and characteristics. Thus, cervical cancer (CC) and endometrial cancer (EC) are two separate entities with different epidemiologic aspects. The incidence and mortality change throughout the world for both these tumors; CC has not been equally prevented in developing and developed countries, and EC is a tumor mainly associated with lifestyle factors contributing as risk factors—being overweight, adulthood obesity, and body fat distribution.

EC is the most frequent gynecological cancer, with an estimated 67,880 new cases in the US in 2024 [8]. This cancer should not be considered a single entity but a group of heterogeneous diseases, as demonstrated by the recent update of FIGO staging, incorporating histological and molecular features considered fundamental for appropriate prognostication [9]. In this regard, histological type is divided into low- and high-risk groups, tumor grade, myometrial invasion, lymphovascular space invasion (LVSI), cervical stromal invasion, adnexal involvement, uterine serosal involvement, lymph node status, as well as molecular classification, including the assessment of POLE mutation, mismatch repair deficiency, non-specific molecular profile, and p53 abnormalities, are of pivotal importance for prognostication. Tumors with POLE mutations generally have an excellent prognosis due to their high responsiveness to treatment and low recurrence rate. On the other hand, tumors characterized by p53 abnormalities often indicate a poor prognosis, associated with higher aggressiveness and lower survival rates. Mismatch repair-deficient tumors, often associated with Lynch syndrome, present an intermediate prognosis. Non-specific molecular profile tumors also show variable outcomes, typically falling between the prognoses of POLE-mutated and p53-abnormal tumors. Recognizing imaging biomarkers for different mutations thus becomes crucial given the significant prognostic differences these mutations have.

However, most of the abovementioned biomarkers relate to the availability of pathological specimens from biopsies or from the uterus removal.

CC is the third most frequent gynecological cancer, with estimated 13.960 new cases in the US in 2023 [10]. Currently, no validated biomarkers are widely available for the diagnosis, prognosis, and follow-up of patients affected by cervical cancer. Imaging evaluation is usually performed for pre-treatment staging and for follow-up, according to the most recent guidelines [11, 12]. Therefore, many imaging biomarkers are also under evaluation for better prognostication of CC.

The purpose of this review is to summarize the available published data about the current possible applications of imaging biomarkers in staging, treatment response evaluation and prognostication, in EC and CC.

## Uterine malignancy treatment strategies

Early-stage CC (IA-IB) is usually treated by surgery, whereas in those cases that have progressed to locally advanced disease, encompassing II-IVA stages, the protocol consists of cisplatin-based chemotherapy plus concurrent radiation. For metastatic, progressive, or persistent disease, systemic treatment is the most adequate therapeutic choice. The standard chemotherapeutic regimen is platinum-based chemotherapy. Tewari et al showed that anti-angiogenic agent bevacizumab significantly improved overall survival of patients affected by metastatic, persistent, or recurrent cervical carcinoma [13]. Moreover, new ESGO-ESTRO-ESP guidelines [14] introduced the use of the immune checkpoint inhibitor Pembrolizumab associated with platinum-based chemotherapy (carboplatin/cisplatin plus paclitaxel) ± bevacizumab in chemo-naive recurrent/metastatic disease with PD-L1 positivity [15]. Another checkpoint inhibitor, the PD1 inhibitor cemiplimab, has been approved for the treatment of patients who progressed after first-line platinum-based chemotherapy regardless of the PD-L1 status if they had not already received immunotherapy [16]. Finally, recent evidence suggests adopting Pembrolizumab associated with chemo-radiation for locally advanced disease with major benefit being driven by patients with FIGO stage III-IVA disease [17].

Regarding EC, the treatment consists of surgery in most of the cases. Adjuvant treatment may vary based on histology, spreading and molecular characteristics. Since conventional pathologic analysis presents consistent prognostic limitations, a new molecular classification is completely changing the therapeutic algorithm of this tumor. This categorization has been introduced based on clinical data reported by different groups that have applied a diagnostic algorithm using three immunohistochemical markers (p53, MSH6, and PMS2) and one molecular test (mutation analysis of the exonuclease domain of POLE) to identify prognostic groups analogous to the TCGA molecular-based classification published in 2013 [18]. Recently, the International Federation of Gynecology and Obstetrics (FIGO) proposed a revised staging approach to this tumor, stressing the importance of introducing the addition of molecular subtype evaluation to the staging criteria as it allows a better prediction of prognosis classes of risk, even if this new staging system is still under validating confirmation. In substance, according to ESGO-ESTRO-ESP guidelines, the therapeutic approach depends on risk class mainly consisting of:

- observation in low risk
- adjuvant brachytherapy in intermediate and high-intermediate risks to reduce the possibility of local recurrence
- external beam radiotherapy after surgery with optional additional adjuvant chemotherapy (especially for high-grade and/or substantial LVSI in high intermediate risk) or with concurrent and adjuvant chemotherapy or alternatively sequential chemotherapy and radiotherapy in high risk (especially for substantial LVSI and/or for stage II tumors)
- chemotherapy alone as an option for high-risk tumors or for advanced disease.

Finally, several anti PD-1 and anti PD-L1 checkpoint inhibitors as monotherapy or in combination have been shown to have activity in advanced, metastatic, or recurrent disease, particularly in microsatellite unstable (microsatellite instability-high) tumors [19–23].

Standardization of quantitative response criteria in uterine cancer treatment is crucial for evaluating treatment efficacy consistently across different studies and practices. Current methodologies vary, leading to challenges in comparing outcomes. For instance, MRI is used to assess changes in tumor size and morphology after treatment, but discrepancies in imaging protocols can result in variable assessments of tumor response [24, 25]. Defining the optimal timing for these assessments is also critical, as early imaging might predict long-term outcomes [26]. Moreover, the thresholds used to define response, such as changes in tumor size or imaging parameters like the apparent diffusion coefficient (ADC), need clear justification based on clinical outcomes, and these thresholds can vary significantly between different studies [25, 27]. Integrating multiple imaging parameters, including multiparametric MRI and advanced techniques like intravoxel incoherent motion (IVIM) and dynamic contrast-enhanced (DCE)-MRI, could lead to more accurate and comprehensive assessments. These methods provide insights into both the physiological and anatomical aspects of tumors, enhancing the prognostic value of imaging biomarkers [28–30].

## Research criteria

This narrative review aims to provide an up-to-date overview of recent scientific evidence on imaging biomarkers in uterine cancers. The search was conducted by consulting three databases—PubMed, Web of Science and Scopus—using keywords such as (endometrial cancer) OR (cervical cancer), including synonyms (tumor, carcinoma, …) AND (dynamic contrast-enhanced MRI) OR (DWI) OR (spectroscopy). Data extraction was done independently by two reviewers; any disagreements were resolved by discussion with a third reviewer.

English-language articles published between January 2001 and November 2024 were considered; the search was further expanded by examining the references of retrieved articles to identify additional potentially suitable studies.

The selection of articles was based on the relevance of the topic, without following a strict inclusion/exclusion process. As this is not a systematic review, the method used may have limitations related to subjectivity in source selection.

From the articles obtained through our literature search, we selected studies that provided comprehensive descriptions of pathology and imaging methods. Editorial comments, conference abstracts, and short communications were excluded. Following an initial screening of titles, topics, and methods sections, articles that did not align with the objectives of our review were excluded. Subsequently, articles presenting opinions, viewpoints, or anecdotal evidence were also excluded. Our initial literature search yielded about 163 articles; subsequently, 55 were deleted based on the above criteria. Finally, 108 published articles were considered for this review (Table 1).

**Table 1:**
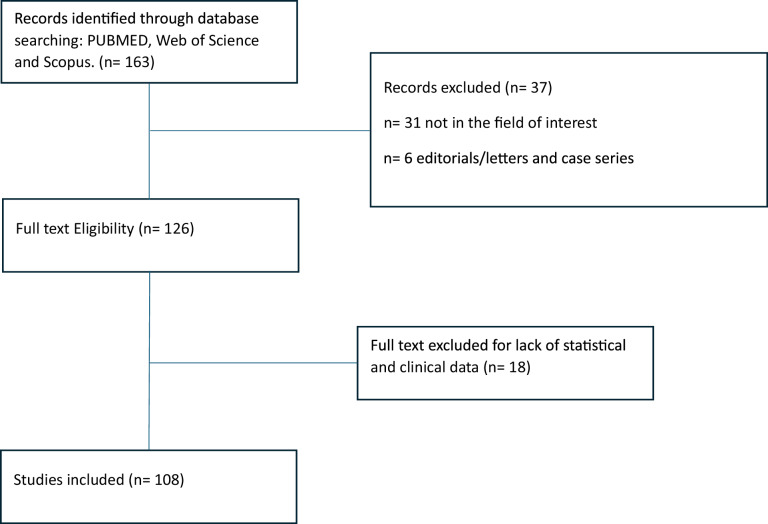
Review flow diagram

## MRI protocol and optimized sequences

Acquiring clear images in a pelvic examination, particularly for small structures, can be challenging. Proper patient preparation and the adoption of an appropriate protocol are crucial to minimizing motion and common artifacts (Table 2). General recommendations for optimizing image quality include the use of an antiperistalsis agent to decrease bowel and uterine peristalsis, ensuring the removal of rectal air, and following a precise protocol for prostheses to prevent distortion artifacts. These measures are essential for enhancing the overall quality of the images [31].

**Table 2:** Suggested MRI protocol for the study of EC and CC

	T2 sagittal	T2 axial	T2 coronal	DWI-ADC	DWI-high b	DCE	T1 3D axial	MRS
Sequence	TSE	TSE	TSE	EPI	EPI	GRE-Twist	VIBE	SVS_SE
TE (ms)	110 (100–140)	134 (100–140)	134 (100–140)	70 (< 80)	72 (< 80)	1.5 (min TE)	2.46/3.69*	30
TR (ms)	9000 (7000–9000)	8000 (7000–9000)	7000 (7000–9000)	5700 (> 3000)	11,500 (> 3000)	4.5 (min TR)	5.6 (5–6)	1500
TF	25 (20–25)	25 (20–25)	25 (20–25)	-	-	-	-	-
FA (°)	160 (150–180)	160 (150–180)	160 (150–180)	-	-	20 (18–20)	9 (8–10)	90
Slice thickness (mm)	3.0	3.0	3.0	3.0	1.8	3.5	0.9	12
Gap (mm)	0	0	0	0.9	0	0	0	-
AVG	2	2	2	-	-	-	1	300
Voxel size (mm^3^)	0.6 × 0.6 × 3.0	0.6 × 0.6 × 3.0	0.6 × 0.6 × 3.0	2.2 × 2.2 × 3.0	1.8 × 1.8 × 1.8	1.4 × 1.4 × 3.5	0.9 × 0.9 × 0.9	12 × 12 × 12
b-values s/mm^2^	-	-	-	0 (2avg), 500 (4avg), 1000 (6avg)	2000 (8avg)	-	-	-
Acquisition time (m:s)	3:50	3:20	1:55	4:18	5:34	0:6.8 (20 meas)	2:27	7:36

* Optimal values for In-phase and out-phase acquisition at 3 T

For our protocol setup, the examination is performed with the patient lying in the supine position using a 3-T MRI scanner. We employ a single body matrix coil in conjunction with a spine array coil. Although an endorectal or endovaginal coil could be used to improve image quality further, we have opted not to utilize these in this case to maintain patient comfort and streamline the procedure [32].

Our choice of a 3-T MRI scanner (MAGNETOM Vida, Siemens Healthineers, Erlangen, Germany) provides high-resolution images, which are particularly beneficial for visualizing small pelvic structures. The higher field strength enables faster scan times and enhances functional techniques like diffusion-weighted imaging (DWI) and dynamic contrast-enhanced imaging, providing more reliable diagnostic data. Furthermore, the increased contrast-to-noise ratio (CNR) at 3 T facilitates better differentiation of tissue interfaces, aiding in the detection and staging of pelvic cancers such as those of the cervix, endometrium, and prostate. The combination of a body matrix coil and a spine array coil allows for comprehensive coverage and optimal signal reception. This setup strikes a balance between image quality and patient comfort, making it suitable for a broad range of clinical scenarios. This setup ensures adequate signal coverage across the pelvic region while maintaining a high signal-to-noise ratio (SNR). The close proximity of the body matrix coil to the region of interest significantly enhances signal reception, while the spine array coil contributes to uniform signal acquisition, especially for deeper structures.

Advanced imaging methods, such as IVIM and magnetic resonance spectroscopy (MRS), also benefit from improved SNR and metabolite peak resolution at 3 T, aiding in tumor characterization and treatment monitoring. Challenges like susceptibility artifacts are mitigated through optimized protocols and advanced sequences, making 3-T MRI a powerful tool for accurate and efficient pelvic imaging.

By adhering to these general recommendations and our specific protocol setup, we aim to achieve the best possible imaging outcomes for pelvic examinations.

A morphological phase is the core of the examination, involving the acquisition of T1-weighted and T2-weighted turbo spin echo images. Subsequently, a functional phase takes place, including the acquisition of DWI and dynamic contrast-enhanced (DCE) images. Finally, after the administration of contrast medium, a high-resolution 3D T1 Fat-saturated image is acquired.

### Morphological imaging and dynamic contrast-enhanced (DCE) MRI

**Fig. 1:**
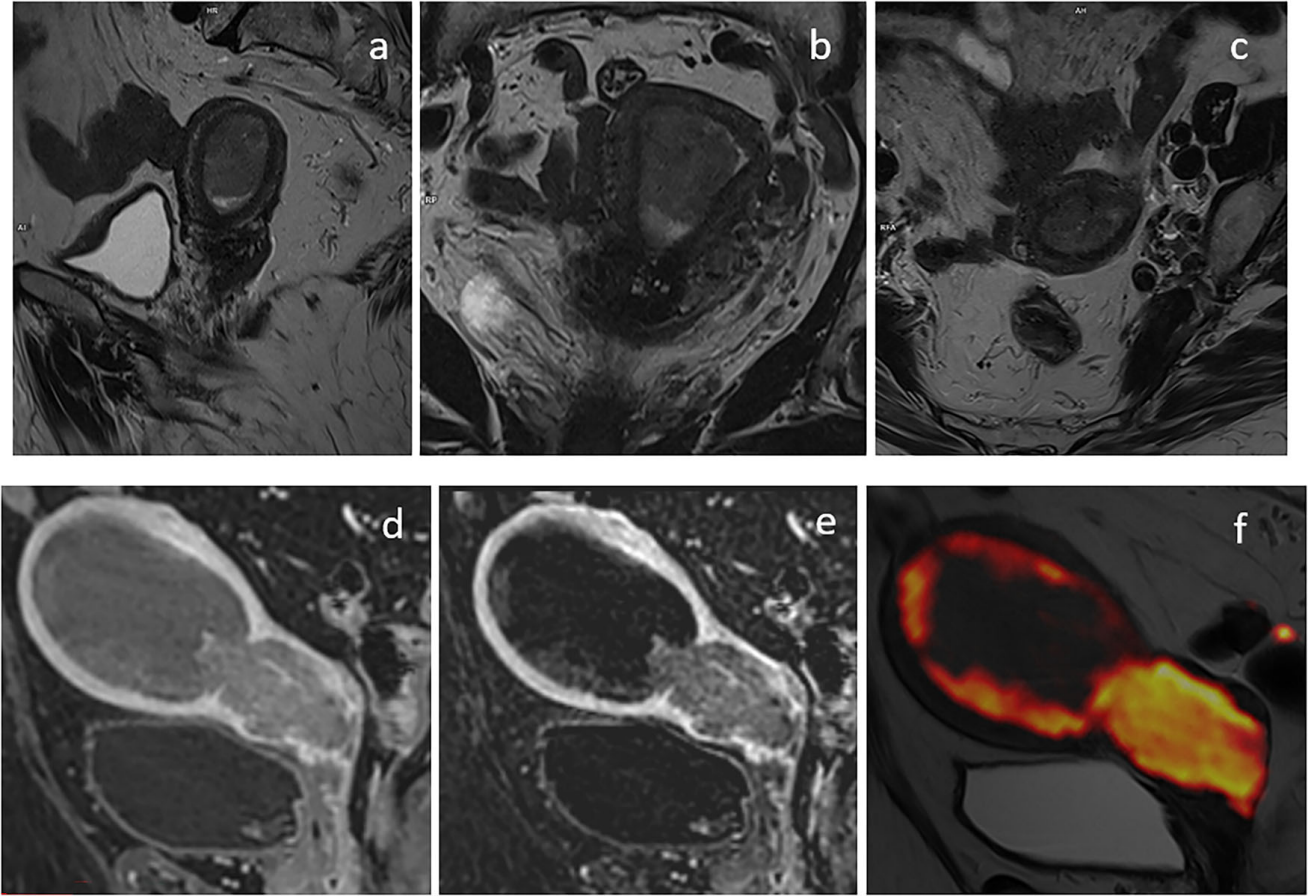
Acquisition on a 67-year-old patient with endometrial cancer for whom images were optimized for optimal contrast, respectively: (**a**) sagittal plane, (**b**) coronal plane, (**c**) axial plane. Acquisition on a 71-year-old patient: **d** post-contrast T1 3D volumetric, **e** subtraction between pre-contrast and post-contrast in T1 3D volumetric, (**f**) fusion between sagittal T2 and isotropic b2000

Due to the natural contrast between the signal intensity of the uterus and the surrounding fat, T2-weighted imaging (T2WI) plays a vital role in pelvic MRI. A high-contrast resolution T2WI can effectively depict all four layers of the cervix [31], requiring a proper combination of parameters. It consists of 3 mm slice thickness, a field-of-view of tailored to the extent of the uterus, while keeping it as small as possible to strike a suitable compromise for resolution (Fig. 1a–c).

In this study, the T2 weighting of the imaging sequence was manipulated by extending the relaxation time and adjusting the Turbo Factor proportionately. To enhance the differentiation among the various layers of the cervix, a deliberate decision was made to keep the echo time at a lower value. This resulted in a grayish appearance of fluids, a characteristic that was corrected using the RESTORE technique.

The high-resolution T1 fat-saturated image utilizes a VIBE sequence with DIXON suppression and a voxel size of 0.9 × 0.9 × 0.9 mm^3^ (Fig. 1d, e).

This approach offers several advantages for pelvic MRI, ensuring precise anatomical evaluation and optimized diagnostic utility. The isotropic voxel size guarantees equal spatial resolution across all three dimensions, facilitating accurate multi-planar reconstructions (MPR) without degradation of image quality. This capability is crucial for the detailed evaluation of small structures and for detecting subtle anatomical or pathological changes, particularly in complex pelvic anatomy. Additionally, isotropic imaging supports advanced post-processing techniques, including 3D segmentation, volumetric analysis, and fusion with other images, such as DWI.

The DIXON technique is optimized by ensuring precise echo time (TE) settings to accurately separate fat and water signals. Multi-echo DIXON is preferable for robust fat suppression in regions affected by field inhomogeneities, such as the pelvis. This results in improved contrast resolution, which is essential for identifying enhancing lesions, delineating tumor margins, and evaluating surrounding tissue structures. Achieving optimal T1-weighted contrast requires fine-tuning of repetition time (TR) and TE. A shorter TR is used to enhance T1 contrast while maintaining a TE that minimizes susceptibility artifacts. The use of gadolinium-based contrast agents can further enhance contrast resolution, particularly for evaluating enhancing lesions and tumor vascularity.

Higher receiver bandwidths reduce chemical shift artifacts and susceptibility-induced distortions, making them particularly beneficial in pelvic imaging. However, excessive bandwidth can lead to noise amplification, requiring careful adjustment. For 3-T MRI systems, attention must be given to mitigate B1 inhomogeneities and dielectric effects, which can impact signal uniformity, especially in deeper pelvic structures.

The DCE sequence employs a VIBE (Volume Interpolated Breath-hold Examination) sequence combined with a TWIST (Time-Resolved Angiography With Stochastic Trajectories) algorithm. This approach optimizes dynamic contrast-enhanced imaging by dividing the k-space into two distinct regions: a central region, which contains low spatial frequency information critical for image contrast, and a peripheral region, which captures high spatial frequency details important for spatial resolution. By prioritizing the acquisition of the central region more frequently than the peripheral one, TWIST achieves a higher temporal resolution without significantly compromising spatial resolution or image quality [33]. To further enhance the optimization of this protocol, parameters such as the sampling density and the temporal footprint of the peripheral region are carefully adjusted. These adjustments are tailored to the clinical requirements, such as the speed of contrast uptake and washout, ensuring that even rapid vascular dynamics are adequately captured. The choice of temporal resolution strikes a balance between capturing the necessary physiological changes and maintaining an adequate SNR.

DCE-MRI is a new functional imaging technique that assesses quantitative physiological parameters associated with tissue microcirculation [34].

The capillary network allows bidirectional exchanges between blood and tissues, and any alteration at this level can be used as an imaging biomarker for diagnosis, prognostic evaluation, and treatment of various diseases, especially in the neoplastic setting [35, 36].

DCE-MRI involves the rapid acquisition of T1-weighted images before, during, and after injection of an intravenous contrast media (CM) (0.1–0.2 mMol/kg of gadolinium-based CM delivered at a flow rate of 2–4 mL/s) [37] and enables the assessment of tissue enhancement kinetics at different time intervals based on the principle that a bolus of paramagnetic low-molecular-weight CM is temporarily confined within the vascular space as it traverses the capillary bed; subsequently, it rapidly transitions into the extravascular extracellular space, commonly referred to as the dispersion space. This transition occurs at a rate influenced by the permeability, surface area, and blood flow of the microvessels [34, 36].

Thus, the dynamics of low molecular weight contrast agents within tissues are determined by three primary factors: blood perfusion, the passage of contrast agents through vessel walls, and the diffusion of contrast media into interstitial space.

In this context, utilizing specialized software employing suitable mathematical models facilitates the extraction of the following parameters associated with tissue perfusion and microvascular status [38, 39]:

- Tissue blood flow: the flow of blood entering (and exiting) a tissue volume.
- Voxel capillary blood volume (Vs): the fraction of blood volume contained within the voxel; also known as blood volume fraction.
- Interstitial volume (Ve): the fraction of extravascular and extracellular volume.
- Ktrans: the rate of transfer from blood to extravascular extracellular space. Ktrans is the most significant perfusion-related parameter in DCE-MRI [40].

It has been shown that tumor perfusion parameters are quantitative biomarkers that reflect the mirable features of neoplasm progression. The ability to trace molecular features and parameters correlated with disease aggressiveness, may represent a breakthrough in the management of many cancers, and allow preoperative risk stratification to guide surgical treatment and adjuvant therapy [34].

To achieve the best results in quantitative MRI analysis, it is essential to standardize imaging protocols across various platforms. This involves using calibration phantoms to ensure that scanner performance is consistently aligned. Additionally, the regular validation of analysis software through the use of digital reference objects is vital for ensuring both accuracy and reproducibility. By maintaining consistent imaging parameters and using the same contrast agents, variability can be greatly reduced, thus enhancing the reliability of the results. These thorough practices of standardization and validation are not only crucial for the success of multi-center studies but also significantly improve the clinical utility of quantitative MRI diagnostics.

In June 2023, the International Federation of Gynecology and Obstetrics (FIGO) updated the staging system for EC by introducing different histologic types, tumor patterns, and molecular classifications [9].

This new system incorporates molecular features and other non-anatomic parameters into clinical consideration and marks a significant departure from traditional staging methodologies that generally neglect histologic and molecular parameters.

Considering the new staging system, DCE-MRI offers the possibility of obtaining quantitative information on physiologic and microstructural aspects associated with disease aggressiveness and facilitating the identification of critical prognostic factors for EC.

Quantitative imaging could represent a major impactful change in the therapeutic course of EC contributing significantly to the staging process, reflecting the complex nature of different histologic subtypes and various underlying biological behaviors.

The use of DCE offers the advantage of being able to assess tissue behavior at different time intervals.

In patients with EC, the myometrial invasion is more readily recognizable during the equilibrium phase of DCE (2 min and 30 s after the administration of the CM).

The subendometrial invasion—recognizable by the presence of an interruption in the enhancement of the subendometrial zone—is better visualized in the earlier phases at around 35–40 s after the administration of the CM. This aspect is of fundamental importance since the exclusion of myometrial invasion allows for conservative therapeutic approaches with fertility preservation.

The clinical management and prognosis of EC are intricately linked to the extent of cervical invasion (CI). CI is deemed present when abnormal signal intensity extends into the cervical canal or stroma, or when the cervical canal displays widening. In DCE-MRI, cervical invasion is characterized by the interruption of the enhancement of the normal cervical epithelium. CI is better assessable in later scans at approximately 4–5 min after the administration of the CM.

DCE-MRI showed superior diagnostic performance compared with T2WI in identifying cervical invasion. Combining T2WI with DCE-MRI demonstrated higher aggregate specificity, positive likelihood ratio, diagnostic odds ratio and area under the curve (AUC) than using T2WI or DCE-MRI alone.

Recently, several authors have highlighted the increasing contribution of DCE-MRI as a source of quantitative biomarkers (Ve, Vs, Ktrans) reflecting progression/recurrence, histologic subtype (aggressiveness) and clinical course in ECs.

Many tumors often exhibit aggressive hypoxia-related biological features [41, 42]: advanced grade endometrial tumors with aggressive features, such as grade 3, advanced FIGO stage, and non-endometrioid subtype show rapid growth (hypercellularity) and contextual areas of necrosis resulting in tissue hypoxia [43, 44].

The dynamic enhancement pattern observed in MRI reflects both tumor blood flow and oxygenation status [45, 46].

Satta et al [47] demonstrated that elevated Ktrans and Ve values are linked to a more favorable prognosis in EC. Their study revealed a statistically significant correlation between Ktrans and Ve values and tumor grading (*p* = 0.01 and < 0.01, respectively). Higher values were associated with well-differentiated G1-G2 tumors (Ktrans: 0.55 ± 0.31 mL/min/100 mL; Ve: 0.31 ± 0.13 mL), whereas lower values were observed in less differentiated G3 tumors (Ktrans: 0.32 ± 0.25 mL/min/100 mL; Ve: 0.18 ± 0.09 mL).

Further studies confirm previous findings: Haldorsen et al [48] observed that diminished tumor blood flow (bf) and lower Ktrans levels were significantly linked to a decrease in the progression-free survival/recurrence ratio, backing the theory that disorganized angiogenesis and hypoxia contribute to the advancement of tumors and the spread of metastases in ECs.

Similarly, Fasmer et al [49] showed that reduced tumor bf and a lower rate constant for contrast agent intravasation (kep) were associated with high-risk histologic subtypes (*p* ≤ 0.04 for both), leading to poorer prognosis (*p* ≤ 0.09).

In a study by Haldorsen et al [44], reductions were observed in bf, E (extraction factor), Vb, Ve, PS, and Ktrans compared to normal myometrium. Non-endometrioid carcinomas (*n* = 12) exhibited lower bf and E levels compared to endometrioid carcinomas (*n* = 43; *p* < 0.05), demonstrating the feasibility of DCE-MRI in reflecting histological subtype and identifying patients at increased risk of recurrence.

Finally, Wang et al [50] explored the contributory value of quantitative parameters obtained from dynamic contrast-enhanced magnetic resonance imaging (DCE-MRI) in distinguishing between TP53 mutant and TP53-wild types, as well as low-risk and non-low-risk early-stage EC, providing a potential reference for the clinical management of early-stage EC.

In the TP53 mutant group (low risk), higher values of Ktrans and Kep were observed compared to the TP53-wild group (non low risk). When identifying early-stage low-risk and non-low-risk EC, Ktrans, Ve, and f emerged as independent predictors, with their combined use demonstrating optimal diagnostic efficacy (AUC, 0.947; sensitivity, 83.33%; specificity, 93.18%). These results are in line with previous studies and suggest a potential association between elevated k trans values in the mutant group with the ability of the TP53 gene to stimulate angiogenesis [51, 52], further indicating the potential role of Ktrans in risk stratification of early-stage EC.

Within the realm of cervical cancer, DCE-MRI assumes a crucial role in prognosticating therapeutic efficacy [53, 54]. This is achieved through the correlation of quantitative measurements with tumor response in CC patients [55, 56].

It is hypothesized that inadequate tumor blood supply, impaired oxygenation status, and the existence of hypoxic cells are determinants of treatment failure (radiotherapy and chemotherapy) [57–59].

DCE-MRI holds promise in potentially predicting early resistance to angiogenesis-related treatments or assessing the response to drugs targeting angiogenesis, as supported by the following research studies [60].

Mayr et al [56] conducted a comparison of dynamic enhancement values in tumors, revealing that patients with advanced CC who exhibited low dynamic enhancement values had a substantially greater likelihood of recurrence compared to those with high dynamic enhancement (78% vs. 0%, *p* = 0.002). Additionally, tumors exhibiting higher mean dynamic enhancement values demonstrated a more favorable response and indicated improved overall blood and oxygen supply.

Parameters including Ktrans-e0, Kep-e0, ΔKtrans, and ΔVe exhibited positive correlations with the tumor regression rate. The average values of Ktrans-e0, Ktrans-e3, ΔKtrans, and ΔVe were elevated in the group without residual tumor compared to the group with residual tumor and emerged as independent prognostic factors for predicting residual tumor occurrence [61].

In conclusion, quantitative imaging could help in distinguishing between benign and malignant cervical tumors, thereby averting unnecessary radical surgery. Song et al [62] illustrated that quantitative parameters such as Kep, Ktrans, and Ve derived from DCE-MRI were markedly superior in the malignant tumor group compared to the benign tumor group (*p* < 0.05). The specificity and sensitivity of Kep, Ktrans, and Ve were notably higher in malignant cervical tumors than in benign ones (Table 3).

**Table 3:** DCE studies conclusions

	Author	Title	Patients	Results
1	Satta	Quantitative diffusion and perfusion MRI in the evaluation of endometrial cancer: validation with histopathological parameters.	44	Ktrans and Ve showed statistically significant correlation with tumor grading (*p* = 0.01 and < 0.01, respectively), indicating that higher values were associated with more differentiated G1-G2 tumors (Ktrans: 0.55 ± 0.31 mL/min/100 mL; Ve: 0.31 ± 0.13 mL) and lower values in less differentiated G3 tumors (Ktrans: 0.32 ± 0.25 mL/min/100 mL; Ve: 0.18 ± 0.09 mL).
2	Haldorsen	Increased microvascular proliferation is negatively correlated to tumor blood flow and is associated with unfavorable outcome in endometrial carcinomas	54	Both low Fb and low Ktrans were significantly linked to decreased progression-free survival/recurrence (*p* < 0.05).
3	Fasmer	Preoperative quantitative dynamic contrast-enhanced MRI and diffusion-weighted imaging predict aggressive disease in endometrial cancer	177	Tumor blood flow (F_b_) and the low rate constant for contrast agent intravasation (kep) were correlated with high-risk histologic subtypes (*p* ≤ 0.04 for both) and exhibited a tendency towards poorer prognosis (*p* ≤ 0.09).
4	Haldorsen	Dynamic contrast-enhanced MRI in endometrial carcinoma identifies patients at increased risk of recurrence—haldorsen	54	Endometrial carcinoma tissue exhibited decreased Fb, E, Vb, Ve, PS, and Ktrans in comparison to normal myometrium. Among non-endometrioid carcinomas (*n* = 12), Fb and E were lower compared to endometrioid carcinomas (*n* = 43; *p* < 0.05).
5	Wang	Quantitative dynamic contrast-enhanced parameters and intravoxel incoherent motion facilitate the prediction of TP53 status and risk stratification of early-stage endometrial carcinoma	74	In the TP53-mutant group, Ktrans and Kep were higher than in the TP53-wild group; Ktrans, Ve, were lower in the non-low-risk group than in the low-risk group (all *p* < 0.05). In identifying early-stage low-risk and non-low-risk EC,.
6	Mayr	Pixel analysis of MR perfusion imaging in predicting radiation therapy outcome in cervical cancer	16	Advanced cervical cancer patients with low dynamic enhancement values have a significantly higher probability of tumor recurrence than those with high dynamic enhancement (78% vs. 0%, *p* = 0.002)
7	Liu	DCE-MRI quantitative parameters as predictors of treatment response in patients with locally advanced cervical squamous cell carcinoma underwent CCRT	48	K^trans^-e0, K_ep_-e0, ΔK^trans^, and ΔV_e_ were positively correlated with the tumor regression rate. Mean values of K^trans^-e0, K^trans^-e3, ΔK^trans^, and ΔV_e_ were higher in the non-residual tumor group than the residual tumor group and were independent prognostic factors for predicting residual tumor occurrence.
8	Song	Analysis of quantitative and semi-quantitative parameters of DCE-MRI in differential diagnosis of benign and malignant cervical tumors	51	Kep, Ktrans and Ve in the malignant-tumor-group were critically higher than that in the benign tumor group (*p* < 0.05). When distinguishing between the benign and malignant cervical tumors, the specificity and sensitivity of kep, Ktrans and Ve were higher in the differential diagnosis of malignant cervical tumors than in the benign cervical tumors.

### T2 mapping

T2 mapping is an advanced MRI technique that quantitatively measures the transverse relaxation time of tissues, providing a detailed characterization of tissue properties. This quantitative imaging tool is especially useful in the context of uterine tumors, where conventional MRI relies heavily on subjective interpretation by radiologists.

The utility of T2 mapping extends to differentiating various uterine conditions without the need for contrast agents, making it a safer option for patients who may be allergic to gadolinium or have renal insufficiency. Studies have shown that T2 mapping can effectively discriminate between benign and malignant uterine lesions, such as differentiating adenomyosis and myoma from cervical and endometrial cancers by analyzing the variation in T2 relaxation times [63–65].

Furthermore, T2 mapping has been shown to enhance the preoperative assessment of endometrial carcinoma, offering valuable insights into tumor grading and the extent of myometrial invasion, which are critical for treatment planning [65].

### Diffusion-weighted imaging (DWI) applications

DWI is an advanced MRI technique that provides unique insights into the microscopic motion of water molecules within tissues. It measures the random motion of water molecules, known as diffusion, which can be affected by tissue microstructure and pathology. In highly cellular tissues (e.g., tumor tissue), the tortuosity of the extracellular space and cell membrane density limit the apparent diffusion of water molecules. In contrast, cystic and necrotic tissues have fewer barriers to water diffusion and the apparent diffusion is relatively free [66].

A significant optimization was performed for the diffusion-weighted sequence, with two series acquired to meet specific diagnostic objectives. For the calculation of the ADC, b-values of 0, 500, and 1000 s/mm² were chosen. This range was selected because b-values below 1200 s/mm² are optimal for accurately estimating ADC, balancing signal decay and image quality while minimizing noise and motion artifacts. A high b-value of 2000 s/mm² was also acquired but excluded from ADC calculations. Instead, this high b-value was specifically utilized to enhance the visualization of damaged or pathological areas by suppressing signals from normal tissues and fluids, which remain visible at lower b-values.

The 2000 s/mm² sequence was acquired using an isotropic voxel size of 1.8 × 1.8 × 1.8 mm³. This isotropic resolution was selected to facilitate the precise fusion of hyperintensity values from diffusion imaging with the T2-weighted morphological sequence, ensuring better anatomical correlation and improved delineation of lesions.

This combination of b-values leverages the strengths of DW-MRI for evaluating gynecological malignancies, enabling detailed assessment of tissue microstructure for initial detection, localization, and monitoring of treatment response.

Different DW-MRI applications in the assessment of cervix and endometrium cancers are found in the literature, ranging from initial detection and localization to treatment response monitoring. The ability of diffusion models to provide functional information about tissue microstructure makes diffusion a valuable tool in the comprehensive evaluation of these gynecological malignancies.

In quantitative diffusion studies, the ADC is quantified.

In CC investigations, ADC value has been investigated both as a prognostic factor [67–69] and for the prediction of response to therapy and local recurrence detection [70–72]. Lower values of ADC compared to the surrounding cervical tissue were associated with CC area and low ADC was associated with higher risks of tumor recurrence.

The ADC value is obtained using a mono-exponential fit to DWI data acquired using at least one b-value and b = 0 s/mm^2^. Therefore, the ADC value that quantifies diffusion can be partly biased by the perfusion parameters, which are quantified at low b-values (0–150 s/mm^2^ in tissues) [73]. Differently from mono-exponential DWI, intravoxel incoherent motion (IVIM) distinguishes the diffusion of water molecules in the extracellular space from capillary micro-perfusion. Using a bi-exponential model to fit diffusion signal decay at different b-values, three quantitative parameters were quantified: the diffusion (D) that quantifies the true diffusion of water molecules in the extracellular space; the pseudo-diffusion (D*) that quantifies the movement of blood water molecules in the capillary network; and the perfusion fraction (fp) representing the volume percentage of water flowing in the capillaries [74].

The choice of b-values is a critical factor influencing the quantification of IVIM parameters alongside the SNR of each DWI in the IVIM protocol. A balance is generally sought between adequate SNR, a sufficient number of b-values, and a reasonable scan time. The b-values should also be optimized according to the tissue type being analyzed.

For highly perfused tissues, b-values equal to 0, 10, 30, 50, 75, 100, 200, 400, 700, 1000 s/mm² are typically used, with at least six b-values under 150 s/mm² to effectively estimate perfusion parameters [75]. For tissues with normal or poor perfusion, such as tumor tissue characterized by hypercellularity, b-values exceeding 1000 s/mm² are necessary to accurately capture slow water dynamics in carcinomas.

Specific protocols vary by application: for CC investigation, a commonly used IVIM protocol includes b-values of [76] 0, 10, 20, 30, 50, 75, 100, 150, 300, 500, 800, and 1000 s/mm², while for EC, the protocol includes 0, 30, 50, 150, 500, 800, 1000, and 1500 s/mm² [47]. Additionally, shorter scan time protocols, such as 0, 25, 100, 175, 200, 1000 s/mm², have shown no significant differences in parameter quantification compared to protocols with double the number of b-values [76].

In recent years, some authors have investigated IVIM parameters concerning cervical cancer, with major interest focused on discriminating between CC and healthy tissue [77–79], showing that tissue D and fp were significantly lower in cervix cancer than normal tissue. Some studies were performed for the prediction of lymph node metastasis [80] and for the response to concurrent chemo-radiation therapy [81]. Wang et al showed that the D and ADC values were all significantly higher for the responders than for the nonresponders, but no significant differences were observed in the D* and f values [82]. A recent study investigated whether quantitative parameters obtained from IVIM model at baseline MRI correlated with histological parameters and response to neoadjuvant chemotherapy in patients with locally advanced cervical cancer (LACC) [83]. Authors highlighted that D showed significantly higher values in good responder patients and in moderate/high tumor-infiltrating lymphocytes, while fp showed significantly higher values in squamous cell tumors.

Concerning EC investigation, several authors highlighted the growing contribution of diffusion MRI as a source of quantitative biomarkers [84, 85], and some correlation studies between quantitative MR data and prognostic factors have been performed using DWI and IVIM model. Some of these reported results on ADC values to discriminate grading in EC, in particular showing lower ADC values in Grade 3 EC [86, 87]. In particular, ADC can identify tumors with worse prognosis. Lower mean ADC values were observed in patients with non-endometrioid histological type, patients with poorly differentiated tumors (Grade 3), or in more advanced stages (FIGO stage III/IV) [47]. The IVIM model, able to distinguish true diffusion from microcirculation-related perfusion without the use of contrast medium [74], showed promising results in correlating with different risk classes in EC [88]. Moreover, significantly higher D* was found in the endometrioid subtype, negative lymph nodes, and stage IA. The absence of lymphovascular invasion was associated with higher fp values [88].

The limitations of IVIM diagnostics are due to the lack of standardization of the fitting procedure of the IVIM bi-exponential function and the lack of works attesting the reproducibility of the method. Quality control measures are based on the comparison between the diagnosis of CC and EC using IVIM parameters and histological or conventional clinical analysis. Through the correlation and comparison between the structural and physiological parameters extracted from the histological examination and the values of the IVIM parameters, the correlation between the IVIM parameters and the molecular subtypes is obtained [47]. In general, as hypercellularity increases, the D value decreases and generally a very low D value is a negative prognostic factor [83]. Some authors have highlighted threshold values to discriminate different types of tumors [76].

Differently from the diagnosis and prognosis of tumors in other organs [89–92], diffusion tensor imaging (DTI) which allows the evaluation of mean diffusivity (MD) and diffusional fractional anisotropy (FA) has not shown its usefulness in the diagnosis and prognosis of tumors in the cervix and endometrium (Table 4).

**Table 4:** Diffusion studies conclusions

	Author	Title	Patients	Results
1	Lee	Perfusion and diffusion characteristics of cervical cancer based on intraxovel incoherent motion MR imaging—a pilot study.	33	Cervical cancer had the lowest *f* (14.9 ± 2.6%) and was significantly different from normal cervix and leiomyoma (*p* < 0.05). The *D* (0.86 ± 0.16 × 10^−^^3^ mm^2^/s) was lowest in cervical cancer and was significantly different from normal cervix and myometrium (*p* < 0.05) but not leiomyoma.
2	Wang	Comparative study of methods for determining intravoxel incoherent motion parameters in cervix cancer.	30	No significant difference was found between IVIM parameters derived from the segmented method with b-value cutoff of 200 s/mm^2^ and the simultaneous fitting method (*p* > 0.05). Tissue diffusivity (D) and perfusion fraction (f) were significantly lower in cervix cancer than normal tissue (*p* < 0.05).
3	Song	A comparative study of four diffusion-weighted imaging models in the diagnosis of cervical cancer.	46	All parameters except pseudo-diffusion coefficient (D*) differed significantly between cervical cancer and normal cervical tissue (*p* < 0.001).
4	Perucho	Diffusion-weighted magnetic resonance imaging of primary cervical cancer in the detection of sub-centimeter metastatic lymph nodes.	50	Out of twenty-one patients, pelvic lymph node (PLN) involvement was observed in 21 cases, with 10 exhibiting metastatic PLNs measuring less than a centimeter. As nodal status progressed from no malignant involvement to sub-centimeter and then size-significant PLN metastases, there was a significant increase in DTV (*p* = 0.013), alongside significant decreases in ADC (*p* = 0.015) and f (*p* = 0.006). IVIM proves valuable in discerning PLN involvement; however, its additional utility diminishes with reader experience.
5	Zhang	The value of DWI in predicting response to synchronous radiochemotherapy for advanced cervical cancer: comparison of three mathematical models.	84	The ADC, D or DDC value was lower in responders than in nonresponders groups (*p* = 0.03, 0.02, 0.01).
6	Wang	Assessing the early response of advanced cervical cancer to neoadjuvant chemotherapy using intravoxel incoherent motion diffusion-weighted magnetic resonance imaging.	42	At all three time points, the responders consistently exhibited significantly higher D and ADC values compared to the nonresponders, while no notable variances were detected in the D* and f values. Furthermore, the α value was notably greater in the responders group than in the nonresponders group (*p* = 0.03).
7	Dolciami	Intravoxel incoherent motion (IVIM) MR quantification in locally advanced cervical cancer (LACC): preliminary study on assessment of tumor aggressiveness and response to neoadjuvant chemotherapy.	20	D showed significantly higher values in GR patients (*p* = 0.001) and in moderate/high TILs (*p* = 0.018). Additionally, Fp demonstrated significantly increased values in squamous cell tumors (*p* = 0.006).
8	Li	Intravoxel incoherent motion diffusion-weighted MRI in patients with breast cancer: correlation with tumor stroma characteristics	77	The average D and f values exhibited reductions in stroma-poor tumors compared to stroma-rich tumors (*p* = 0.012, 0.015)Additionally, the mean D value was observed to be lower in the collagen-dominant type compared to both fibroblast-dominant and lymphocyte-dominant types (*p* = 0.032, 0.043).
9	Yan	Can the apparent diffusion coefficient differentiate the grade of endometrioid adenocarcinoma and the histological subtype of endometrial cancer?	98	The mean ADC values (ADCmean) for high-grade endometrioid adenocarcinomas were significantly lower than the values for low-grade tumors (0.800 versus 0.962 × 10^–3^ mm^2^/s) (*p* = 0.002). However, no significant differences in ADCmean and minimum ADC values (ADCmin) were found between tumor grades (G1, G2, and G3) of endometrial cancer.
10	Kishimoto	Endometrial cancer: correlation of apparent diffusion coefficient (ADC) with tumor cellularity and tumor grade.	30	The average ± standard deviation (SD) ADC value (× 10^−^^3^ mm^2^/s) for endometrial cancer was 0.85 ± 0.22 (range, 0.55–1.71). Additionally, the mean ± SD tumor cellularity was 528.36 ± 16.89 (range, 298.0–763.6). Notably, ADC values exhibited a significant inverse correlation with tumor cellularity.
11	Woo	Histogram analysis of apparent diffusion coefficient map of diffusion-weighted MRI in endometrial cancer: a preliminary correlation study with histological grade	33	The standard deviation, quartile, 75th, 90th, and 95th percentiles of ADC demonstrated notable variances among grades (*p* ≤ 0.03 for all) and between high and low grades (*p* ≤ 0.024 for all).
12	Satta	Quantitative diffusion and perfusion MRI in the evaluation of endometrial cancer: validation with histopathological parameters	44	ADC exhibited a notable increase in endometrioid histology, G1-G2 (low grade), and stage IA. Moreover, significantly elevated D* values were observed in the endometrioid subtype, negative lymph nodes, and stage IA.
13	Zhang	Multi-b-value diffusion-weighted imaging for preoperative evaluation of risk stratification in early-stage endometrial cancer	53	The ADC and D values exhibited significant decreases in intermediate-risk compared to low-risk (*p* = 0.000 and 0.011), as well as in high-risk compared to low-risk of early-stage EC (*p* = 0.001 and 0.013), whereas f values only displayed significant distinctions between low-risk and intermediate-risk groups (*p* = 0.011).

### Magnetic resonance spectroscopy (MRS)

MRS is a noninvasive MR method that can be employed to monitor metabolism alterations that are associated with tumor progression or with early drug target modulation and can be predictive of cancer response to treatment. In fact, metabolic alterations due to tumor progression or therapy response occur much earlier than tumor shrinkage, making MRS an optimal tool for tumor characterization and its response to therapy.

**Fig. 2:**
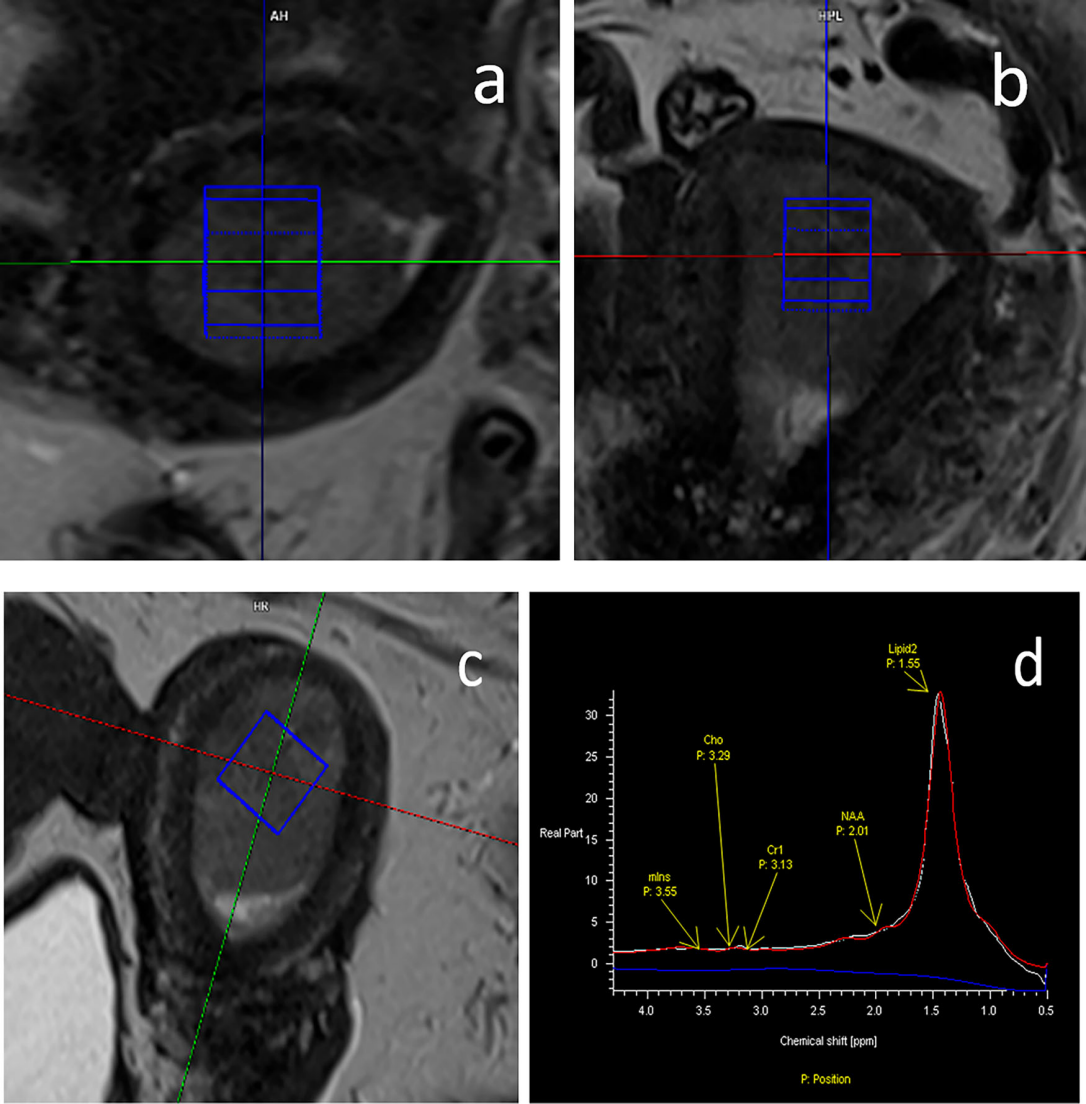
An example of planning of a 12 × 12 × 12 mm^2^ voxel for spectroscopy in three planes (**a** axial, **b** coronal, **c** sagittal) and acquired spectrum (**d**). Voxel size is chosen in homogeneous tumoral areas avoiding hemorrhagic and necrotic regions

MR spectroscopy may be performed by using single- or multivoxel techniques. The PRESS sequence is the most utilized technique for single-voxel selection, but recent consensus papers recommend substituting the PRESS with the semi-Laser technique to minimize the chemical shift displacement error [93] (Fig. 2).

Peak area measured by MRS is proportional to the metabolite concentration and it allows to accurately quantify metabolites if a proper quantitative protocol is adopted [94].

A recent review describes the MRS techniques most utilized to study uterine malignancies and suggests new strategies to improve MRS diagnostic and prognostic potential [95].

Thanks to the increased availability of 3-T scanner since the 2010s, MRS received clinical attention (consensus) as a routine diagnostic tool in several anatomical sites. Recently, new techniques have reduced the artifacts caused by movement associated with respiration and bowel peristalsis, thus allowing the use of MRS in the abdomen and pelvis. Contamination of signal due to pericervical fat is also limited by outer volume suppression techniques. Nevertheless, its introduction into pelvis diagnostic/prognostic tools is limited because MRS can be performed only in centers equipped with advanced instrumentation and trained personnel. In fact, being a low-sensitive technique, it is necessary to use scanner with high magnetic field (≥ 3 T) and to adopt procedures for local homogenization of the magnetic field, which could require expertise and extra acquisition time.

**Fig. 3:**
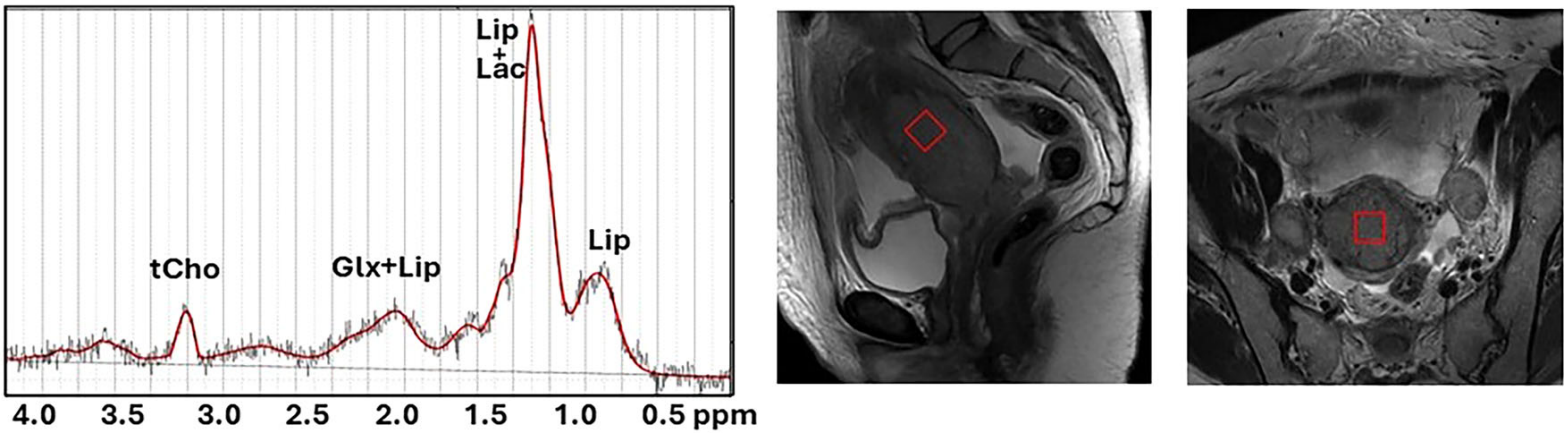
Representative spectrum (TR/TE = 2000/30 ms) acquired in a cervical cancer. The signal comes from the voxel (shown as a red square) positioned in the homogenous tumor region. Peak assignments: Glx, glutamine plus glutamate; tCho, total choline; Lip, lipids; Lac, lactate

Abnormal levels of the signals of choline-containing compounds (with a prominent peak that resonates at 3.2 ppm) or lipids (which present several resonances, among them the highest derives from the methylene group and resonate at 1.3 ppm) are two important contributors in spectra from uterine malignancies [95]. In general, choline is elevated in tumors because choline is a cell membrane component, and increased cell turnover is associated with malignancy. Lipids are a group of molecules that make up the biological membranes and function as energy source and signaling molecules [96]. Lipid accumulation in cancer is caused by many reasons. One of them is metabolic reprogramming, which has been widely observed during cancer development to confer cancer cells the ability to survive and proliferate [97]. Lipid signal is receiving increasing attention in gynecological cancer as a prognostic marker (Fig. 3).

In vivo and ex vivo MRS studies showed high lipid signal at 1.3 ppm as a diagnostic and predictive biomarker for invasive [98] or malignant cervical cancer [99]. Poorly differentiated tumors exhibited more unsaturated fatty acid (by measuring the ratios between the α-carboxyl and α-olefinic groups resonating at about 2.1 ppm over the methyl group resonating at 1.3 ppm) when compared with the well-differentiated tumor or normal tissue [100]. No difference was observed in the choline peak between benign and malignant cervical lesions by several authors [101–103].

The role of MRS for therapeutic response assessment has been investigated by several authors. Reduction in choline signal was observed after radiation therapy [104] but no after non-surgical therapy [105]. Reduction in triglyceride levels has been associated with a reduction in tumor volume in the evaluation of response to neoadjuvant chemotherapy [102].

Elevated lipid resonance helped to predict the poor prognostic HPV genotypes and persistent disease following concurrent chemoradiotherapy in CC [106] and the response to neoadjuvant chemotherapy in patients with LACC [106]. An inverse correlation between Lipid and tCho signal, with lower Lipid and higher tCho in patients who had a good response to NACT was also suggested [106].

MRS seems to potentially aid in the preoperative risk stratification in EC. In fact, tCho signal have been found to be significantly higher in tumor tissue compared with normal myometrial tissue [107]; high tCho associated with high-risk features (tumor volume and grade). Increase of Cho/water signal ratio was found to be associated with increasing Ki67 signal intensity, with deep myometrial invasion (not for cervical invasion) and with lymph node metastasis in estrogen-dependent EC [108].

Interestingly, the acquisition of the spectra with a reasonably short echo time (< 30 ms) makes lipid signal a potential biomarker relevant for risk stratification and prognosis in uterine malignancies.

This research confirms the power of MRS in offering a unique lens for understanding, diagnosing, and assessing treatment response for cervical and endometrial cancer. Limitations in MRS use consist of lower sample sizes and variable acquisition parameters and quantification methods, which make it difficult to generalize the results. Therefore, there is a need to conduct standardized MRS studies to determine the best use for MRS in the diagnostic and prognostic clinical setting for pelvis cancer.

## Conclusions

DCE-MRI provides quantitative biomarkers (Ve, Vs, Ktrans) that reflect disease aggressiveness and critical prognostic factors in endometrial cancer (EC). It outperforms T2WI in detecting cervical invasion and can predict resistance to angiogenesis-related treatments. DW-MRI applications in cervical and endometrial cancers include detection, localization, and treatment response monitoring. IVIM parameters help differentiate cervical cancer from healthy tissue, with lower tissue D and fp values in cancerous tissue. Diffusion MRI, especially DWI and the IVIM model, correlates ADC values with tumor grading and prognosis, where lower ADC values indicate higher-grade tumors and advanced stages. MRS, a noninvasive MR method, monitors metabolic alterations linked to tumor progression or therapy response, occurring earlier than tumor shrinkage. With 3-T scanners and improved techniques reducing motion artifacts, MRS has become routine in abdominal and pelvic diagnostics. Single-voxel MRS captures larger, vital tumor volumes, and correcting for T1 and T2 relaxation times enhances metabolite quantification. Elevated choline and lipid signals in MRS indicate uterine malignancies, with lipid accumulation as a prognostic marker in gynecological cancer.

In conclusion, from the authors’ perspective, integrating techniques such as DCE-MRI, DWI, and MRS significantly enhances their diagnostic and prognostic value in the quantitative imaging of uterine tumors, providing a comprehensive and detailed view essential for effectively guiding therapeutic decisions.

## Data Availability

The data supporting the findings of this review are derived from publicly available sources and published studies, which were identified through a structured search of the PubMed database. All relevant data and materials are referenced within the review. No new data were generated or analyzed during this study. Additional information can be obtained from the corresponding author upon reasonable request.

## Abbreviations

ADC: Apparent diffusion coefficient
AUC: Area under the curve
bf: Blood flow
CC: Cervical cancer
CI: Cervical invasion
CM: Contrast media
D*: Pseudo-diffusion
D: Diffusion
DCE: Dynamic enhancement images
DWI: Diffusion-weighted imaging
E: Extraction factor
EC: Endometrial cancer/carcinoma
FA: Fractional anisotropy
FIGO: International Federation of Gynecology and Obstetrics
fp: Perfusion fraction
IVIM: Intravoxel incoherent motion
LACC: Locally advanced cervical cancer
LVSI: Lymphovascular space invasion
MRI: Magnetic resonance imaging
MRS: Magnetic resonance spectroscopy
SNR: Signal-to-noise
T2WI: T2-weighted imaging
TR: Repetition time
TWIST: Time-resolved angiography with stochastic trajectories
US: Ultrasound
Ve: Interstitial volume
VIBE: Volume interpolated breath-hold examination
Vs: Voxel capillary blood volume
